# Study of soil expansion characteristics in rainfall-induced red-bed shallow landslides: Microscopic and macroscopic perspectives

**DOI:** 10.1371/journal.pone.0246214

**Published:** 2021-01-28

**Authors:** Cuiying Zhou, Guangjun Cui, Hao Yin, Lei Yu, Gankai Xu, Zhen Liu, Lihai Zhang

**Affiliations:** 1 School of Civil Engineering, Sun Yat-sen University, Guangzhou, Guangdong, China; 2 Guangdong Engineering Research Center for Major Infrastructures Safety, Sun Yat-sen University, Guangzhou, Guangdong, China; 3 The University of Melbourne, Parkville, Melbourne, Australia; China University of Mining and Technology, CHINA

## Abstract

The main cause of rainfall-induced red-bed shallow landslides is the tendency of red-bed weathered soil to expand when it meets water. However, studies on the expansion mechanism of expansive soil have not considered the effects of hydration and particle orientation. In this study, the hydration force of soil was determined according to the electric double-layer theory, the particle direction of soil was determined by analyzing images of soil obtained by scanning electron microscopy, and, finally, a microscopic model of the electrical double layer of red-bed weathered expansive soil was established in which the hydration force and soil-particle orientation were taken into account. The results showed that the expansion of red-bed weathered expansive soil is the result of hydration forces and repulsive forces in the electric double layer. The grain orientation of the soil strongly influenced the microscopic model. The unloading expansion rate of red-bed weathered expansive soil decreased with an increase in cation concentration and a decrease in pH value. It increased with an increase in the hydration cation radius. These experiments indicate the reliability of the microscopic model and provide a theoretical basis for the prevention and control of rainfall-induced red-bed shallow landslides.

## 1. Introduction

Red-bed weathered soil is widely distributed in southern China. Because soils of this type contain large amounts of clay minerals such as montmorillonite and illite, they have properties of expansion and contraction. When large amounts of rainfall occur, long-term changes in saturation and pore-fluid chemical changes in red weathering soil can lead to the expansion and fracture of engineering structures, especially due to shallow landslides, which will affect the safety of engineering [1–4]. Although research on the expansive properties of some other expansive soils has been conducted at the microscopic level [5, 6], experimental and theoretical research on red-bed weathered soil is insufficient.

The study of soil expansion properties involves three aspects: theoretical models, experiments and numerical calculations. To understand the expansion mechanism of red-bed weathered soil under water, it is necessary to study the expansion model of expansive soil [7, 8]. The expansion model is composed of the macroscopic elastoplastic constitutive model and the electric double-layer microscopic model. Alonso et al. proposed the Barcelona expansive model for simulating the deformation behavior of expansive soil at both the micro and macro levels [9]. Sánchez et al. and Sheng et al. further improved the Barcelona expansive model by considering plastic behavior in the microstructure and macrostructure systems of bentonite [10, 11]. The Barcelona expansion model is one of many models of soil expansion. Its advantage is that it allows us to analyze the problem from both macro and micro perspectives. Because there are existing research studies on the expansion model [12–14], it will not be described in detail here. The common drawback of these studies is that they all assume that the microcrystalline layers are parallel to each other and do not consider the effect of particle orientation on the soil expansion characteristics. In addition, the effect of hydration force on the expansion is not considered. Both experimental and numerical studies have shown that hydration force is an important factor in the expansion mechanism [15, 16]. The main mechanism through which expansion occurs is that once the hydration cation is adsorbed on the surface of the colloidal particles, the hydration film thickness on the surface of the colloidal particles increases greatly, resulting in expansion of the soil [17]. Gimmi and Churakov studied the distribution of cations and water molecules in flagstone crystal layers using a neutron diffraction test. They found that ionic hydration was the main reason for the expansion of the flagstone crystal layer [18]. Gimmi and Churakov’s findings are consistent with those of Rahromostaqim and Sahimi, who experimentally investigated the hydration energies of various alkali metal ions and the surface hydration energies of montmorillonite [18, 19]. However, these studies are still at the level of experimental or numerical calculation and do not involve the red-bed field; thus, the corresponding theoretical model of expansion of red-bed weathered soil needs to be studied.

Through the above analysis, it was realized that the previous theoretical models, experiments and numerical calculations either did not take into account the influence of hydration force and particle orientation or did not study soils that have the special properties of red-bed weathering soil. Therefore, the purpose of this study was to establish an electrical double-layer microcosmic model of red-bed weathered expansive soils with hydration forces and particle orientation and to verify the validity of the model by a series of experiments. The research described in this paper can provide a theoretical basis for correctly understanding engineering geological problems associated with red-bed weathered expansive soil and for preventing engineering geological disasters such as red-bed shallow landslides and foundation settlement.

## 2. Electric double-layer model of expansive soil with hydration force and particle orientation considered

Unlike ordinary electrostatic forces, the hydration force can be defined as the repulsive force that occurs when two hydrophilic colloidal particles approach each other within a distance of several nanometers [20, 21]. Based on the Gouy-Stern electric double-layer theory, the microexpansion model proposed in this study was developed by integrating the hydrate ion model and the particle orientation distribution. A list of the constants and variables used in the equations is provided in Table 1.

**Table 1:** Constants and variables used in the equations.

Shorthand notation	Constant or variable	Shorthand notation	Constant or variable
*φ*_*s*_	Volume fraction of hydration ions on the Stern layer when the Stern layer is separated	*d*_*min*_	Avoid a lower limit of high-volume fraction of Stern-layer ions
${\hat{\varphi }}_{s}$	Volume fraction of hydration ions on the Stern layer when the Stern layer overlaps	*F*_*s*_	Expansive force between soil particles and layers
*v*_*0*_	Volume of hydrated ions	*F*_*h*_	Hydration force between two particles
*δ*_*0*_	Hydrated cation diameter	*F*_*r*_	Electric double-layer repulsion
*Γ*	Ion adsorption capacity of the particle surface	*F*_*van*_	van der Waals force
*φ*_*s*_*’*	*φ*_*s*_ in terms of the charge density	*u*	Dimensionless potential between two parallel particles
*h*	Distance between two crystalline layers	*H*	Hammett constant
*σ*_1_	Surface charge density of Stern layer	*T*_1_	Thickness of the clay particles or crystal layer
*v*^*+*^	Valence of hydrated ions	*f(θ)*	Function of particle orientation
*${\hat{\varphi }}_{s}’$*	${\hat{\varphi }}_{s}$ in terms of the charge density	*a*, *b*, and *c*	Constant parameters that can be obtained by fitting experimental data; greater than 0
*δF*	Free energy of a tiny volume in a single Stern layer	*θ*	Angle between the particle or layer surface and the horizontal direction
*k*	Boltzmann constant	*F*_*v*_	Vertical component of *F*_*s*_
*T*	Absolute temperature	*F*_*l*_	Transverse component of *F*_*s*_
*A*	Area between two colloidal layers	*Ᾱ*	Average volume within a representative unit of a composite
*n*	Local concentration of ions	*v*	Representative unit volume
*v*_*w*_	Volume of water molecules	*m*	Phase number of particles in soil
*δV*	A tiny volume of a single Stern layer	*v*_*i*_	Volume of phase *i* particles in soil
*F*_*∞*_	Force between the systems when the Stern layer is separated	*F(x)*	Vertical component of the expansive force at any point in the soil
*F*	Force between the systems when the Stern layer overlaps	*c*_*i*_	Volume percentage of phase *i* soil particles
*Δf*_*H*_(*d*)	Free energy of the clay surface system	*F*_*i*_	Vertical expansive force of the *i* phase
*d*	Half the distance between the two crystalline layers		

### 2.1. Electric double-layer model of expansive soil with hydration force considered

Cations generally exist in the form of hydrated ions in aqueous solution. Based on the Gouy-Stern electrical double-layer theory, a hydration force model for expansive soil can be obtained by modifying a protein emulsion system proposed by Paunov et al. [22]. The volume fraction of hydrated ions (*φ*_*s*_) on the Stern layer when the Stern layer is separated and the volume fraction of hydrated ions (${\hat{\varphi }}_{s}$) on the Stern layer when it overlaps can be described as follows [23]:

$$\varphi_{s}=\frac{v_{0}\Gamma }{\delta_{0}}$$ (1)

$$.{\hat{\varphi }}_{s}=\frac{2\delta_{0}}{h}\varphi_{s}=\varphi_{s}+\Delta {\hat{\varphi }}_{s}$$ (2)

The adsorption capacity of a saturated soil system can be described by Henry’s adsorption isotherm [24], but it is not applicable to a mineral colloid system. In this study, the charge density *σ*_1_, which reflects the ion-adsorption capacity of the Stern layer, was introduced to make it possible to derive the volume fraction *φ*_*s*_ of the Stern layer in the clay mineral system. The units in which *σ*_1_ is expressed had to be converted from Coulombs/m^2^ to electron number/m^2^. By dividing *σ*_1_ by the valence of hydrated ions to represent the adsorption capacity of ions on the particle surface, the volume fraction of hydrated ions in the Stern layer can be obtained (Eq 3).

$$\varphi_{s}^{\prime }=\frac{v_{0}\sigma_{1}}{\delta_{0}v^{+}}$$ (3)

If the Stern layer of clay minerals overlaps, the volume fraction of hydrated ions on the Stern layer can be described as

$${\hat{\varphi }}_{s}^{\prime }=\frac{2\delta_{0}}{h}\varphi_{s}^{\prime }=\frac{\delta_{0}}{d}\varphi_{s}^{\prime }=\varphi_{s}^{\prime }+\Delta {\hat{\varphi }}^{\prime }.$$ (4)

The free energy of a tiny volume of a single Stern layer is [22]

$$\delta F=\frac{kT}{v_{w}}(1-\varphi_{s}^{\prime })\text{ln}(1-\varphi_{s}^{\prime })\delta V.$$ (5)

For two separated clay particles with area *A*, the free energy of the system can be derived if the Stern layer is not superimposed. That is,

$$\frac{F_{\infty }}{A}=2\frac{kT}{v_{w}}\int_{0}^{\delta_{0}}\left(1-\varphi_{s}^{\prime }\right)\text{ln}(1-\varphi_{s}^{\prime })dz.$$ (6)

For two overlapping Stern layers, the free energy of the system is

$$\frac{F}{A}=\frac{kT}{v_{w}}\int_{0}^{h}\left(1-{\hat{\varphi }}_{s}^{\prime }\right)\text{ln}(1-{\hat{\varphi }}_{s}^{\prime })dz.$$ (7)

By integrating Eqs 6 and 7, the following equations can be obtained:

$$\frac{F_{\infty }}{A}=\frac{2\delta_{0}kT}{v_{w}}(1-\varphi_{s}^{\prime })\text{ln}(1-\varphi_{s}^{\prime })$$ (8)

$$\frac{F}{A}\approx \frac{2\delta_{0}kT\varphi_{s}^{\prime }}{v_{w}{\hat{\varphi }}_{s}^{\prime }}(1-{\hat{\varphi }}_{s}^{\prime })\text{ln}(1-{\hat{\varphi }}_{s}^{\prime })$$ (9)

Subtracting Eq 9 from Eq 8 and using a first-order Taylor series, the free energy of the clay surface system can be obtained through Eq 10. As Δφ^s'≥0, we obtained Eq 11.

$$\Delta f_{H}(d)=\frac{F-F_{\infty }}{A}=-\frac{2\delta_{0}kT}{v_{w}\varphi_{s}^{\prime }}[\text{ln}(1-\varphi_{s}^{\prime })+\varphi_{s}^{\prime }]\Delta {\hat{\varphi }}_{s}^{\prime }+o(\Delta {\hat{\varphi^{\prime }}}^{2})$$ (10)

$$\Delta f_{H}(d)\approx \left\{ \begin{array}{l} -\frac{2\delta_{0}kT}{v_{w}\varphi_{s}^{\prime }}[\text{ln}(1-\varphi_{s}^{\prime })+\varphi_{s}^{\prime }]\left(\frac{\delta_{0}}{d}-1\right),\;(d_{\min}\leq d\leq \delta_{0})\\ 0,\;(d>\delta_{0}) \end{array}\right.$$ (11)

By taking the derivative of Eq 11 and combining it with Eq 3, the hydration force between two clay particles can be expressed by Eq 12:

$$F_{h}=-\frac{\partial \Delta f_{H}}{\partial d}\approx \left\{ \begin{array}{l} -\frac{2\delta_{0}^{2}kT}{v_{w}d^{2}}[\text{ln}(1-\frac{v_{0}\sigma_{1}}{\delta_{0}v^{+}})+\frac{v_{0}\sigma_{1}}{\delta_{0}v^{+}}],\;(d_{\min}\leq d\leq \delta_{0})\\ 0,\;(d>\delta_{0}) \end{array}\right..$$ (12)

Because the hydration repulsive force and the electric double-layer repulsive force are independent, the repulsive force in the expansive soil can be obtained by summing the hydration force and the electric double-layer repulsive force.

According to the results of Ruckenstein et al. [25], the repulsion of the electric double layer is expressed as

$$F_{r}=2nkT(\cosh u-1.)$$ (13)

According to the Derjaguin–Landau–Verwey–Overbeek (DLVO) electric double-layer model, the van der Waals attraction between expansive soils can be expressed as

$$F_{van}=\frac{H}{6\pi }\left(\frac{1}{{\left(2d\right)}^{3}}+\frac{1}{{\left((2d)+2T_{1}\right)}^{3}}-\frac{2}{{\left((2d)+T_{1}\right)}^{3}}\right).$$ (14)

Therefore, the electric double-layer model of expansive soil with consideration of hydration forces can be established as

$$F_{s}(d)=F_{r}+F_{h}-F_{van}.$$ (15)

### 2.2. Electric double-layer microscopic model of expansive soil considering particle orientation distribution

Current microscopic models of the electrical double layer of expansive soil assume that the clay particle sheets are parallel to each other. In reality, however, the distribution of clay particles is not parallel, and the direction of the expansive force between clay particles is not uniform. In this study, the particle orientation of the soil was quantified by analyzing images of the soil obtained by scanning electron microscopy (SEM).

**Fig 1:**
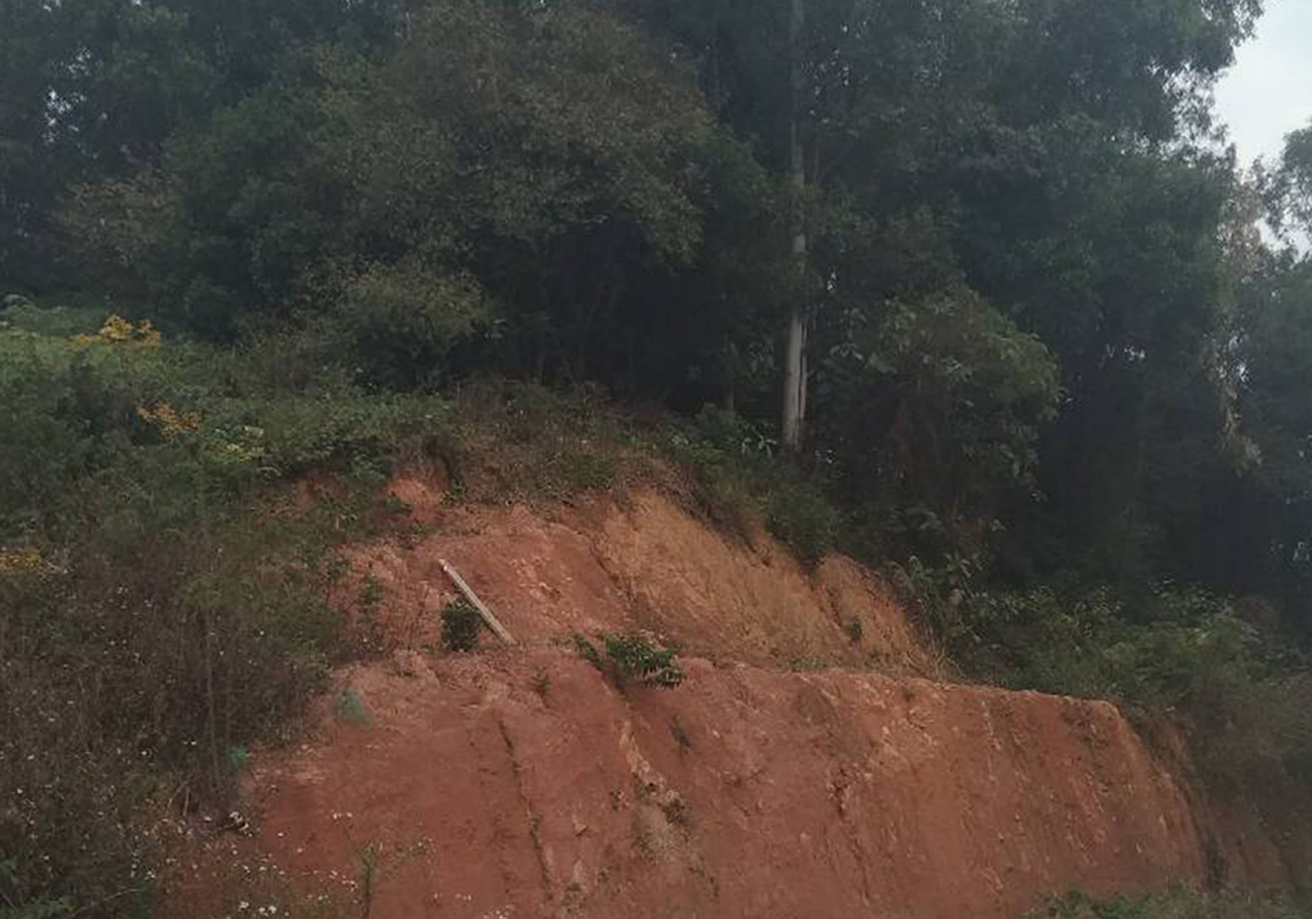
Sampling spot.

The soil samples used in this experiment were taken from the abandoned red-bed slope near the Maoming section of Baotou to Maoming Expressway (Fig 1). The slope is located in the field, is not protected by highway operations, and the sampling site is not located in any private or government restricted area. Therefore, samples can be taken directly from the site without approval from any authoritative organization. The number of samples is small and does not affect the local ecological environment. In addition, the investigation of the red-bed slope is part of the field red-bed investigation of the Guangdong Major Infrastructure Safety Engineering and Technology Research Centre of Sun Yat-sen University. The whole experiment process, including sampling, sample preparation, experiment and other steps, has been approved by Guangdong Major Infrastructure Safety Engineering and Technology Research Centre of Sun Yat-sen University. The instruments and equipment used in this study are all from the Guangdong Major Infrastructure Safety Engineering and Technology Research Centre and the Instrumental Analysis and Research Center of Sun Yat-sen University. We only need to make an appointment to apply for the experiment. Maoming City is located in South China, and its geographical coordinates are 110°19°-111°41°E, 21°22–22°42°N. This city has a subtropical monsoon climate, mountainous and hilly areas and abundant rainfall. The soil samples used in the subsequent macro experiments were taken from the same area. Thirty samples were used in the microscopic study. The dry density of the soil was 1.53 g/cm^3^. Two grams of red-bed weathered soil were taken from each sample and dried in a drying oven for 8 hours. The dried samples were then soaked completely in rain water and allowed to dry naturally for 8 hours. Finally, each individual sample was placed on a sample table containing conductive adhesive, the excess powder was removed, and the sample was sprayed with gold. The EVO-MA10(W) tungsten filament scanning electron microscope (resolution 2–15 nm) at the Instrumental Analysis and Research Center at Sun Yat-sen University was used to detect the mineral composition of and the microstructure of these specimens. The appropriate image analysis position was selected by adjusting the contrast and brightness, the focus knob was adjusted to make the image clearer, and the image was then enlarged 2500 times. The astigmatism was eliminated, and a slow scanning rate was used to obtain the experimental results. The results show that these soils consist mainly of quartz, illite, and kaolinite, with a small amount of montmorillonite. The microstructure of these soils must be studied by image processing techniques.

**Fig 2:**
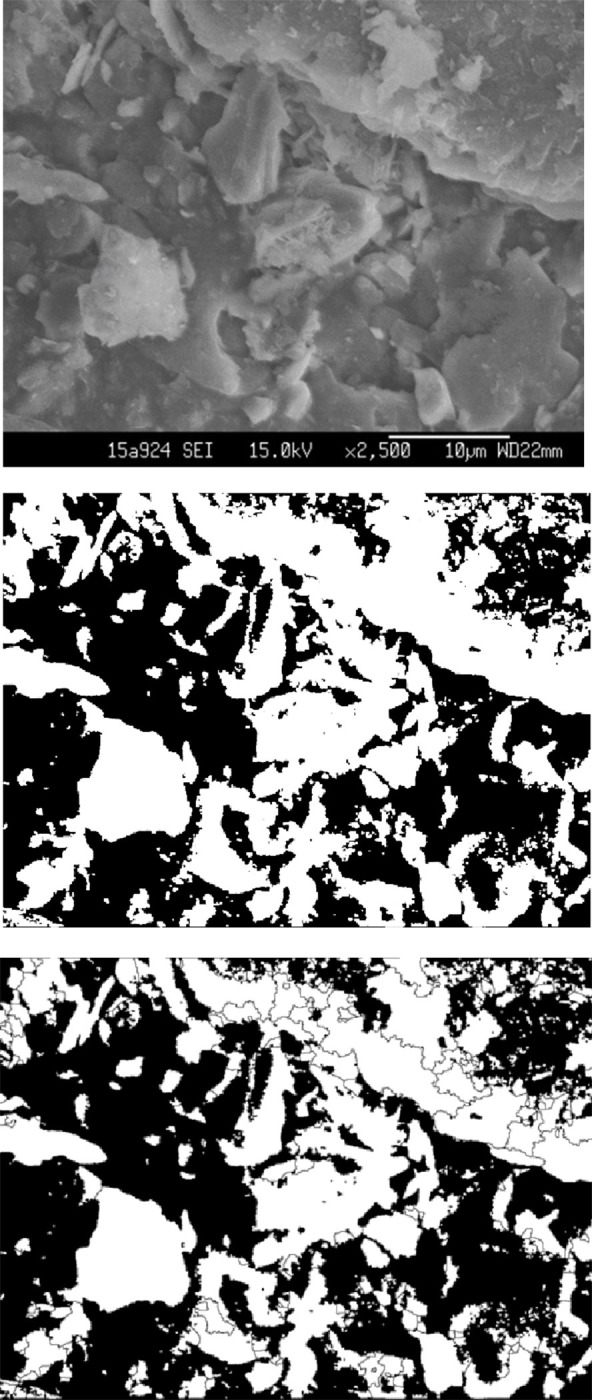
Processing of scanning electron microscopic images: (A) Grayscale images obtained by scanning electron microscopy (2,500×); (B) Binary processing; (C) Particle boundary treatment.

**Fig 3:**
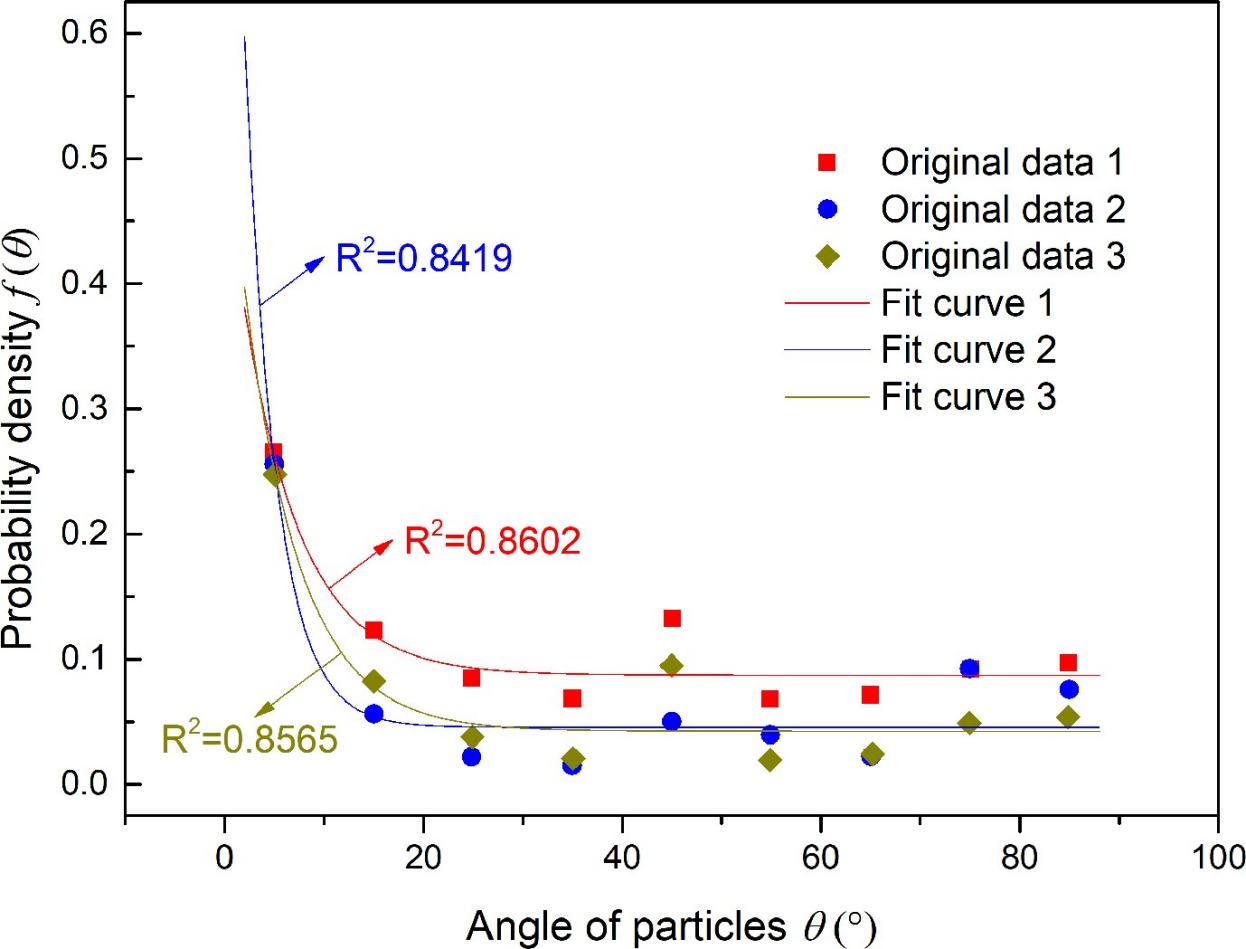
Relationship between particle angle and probability density value.

**Fig 4:**
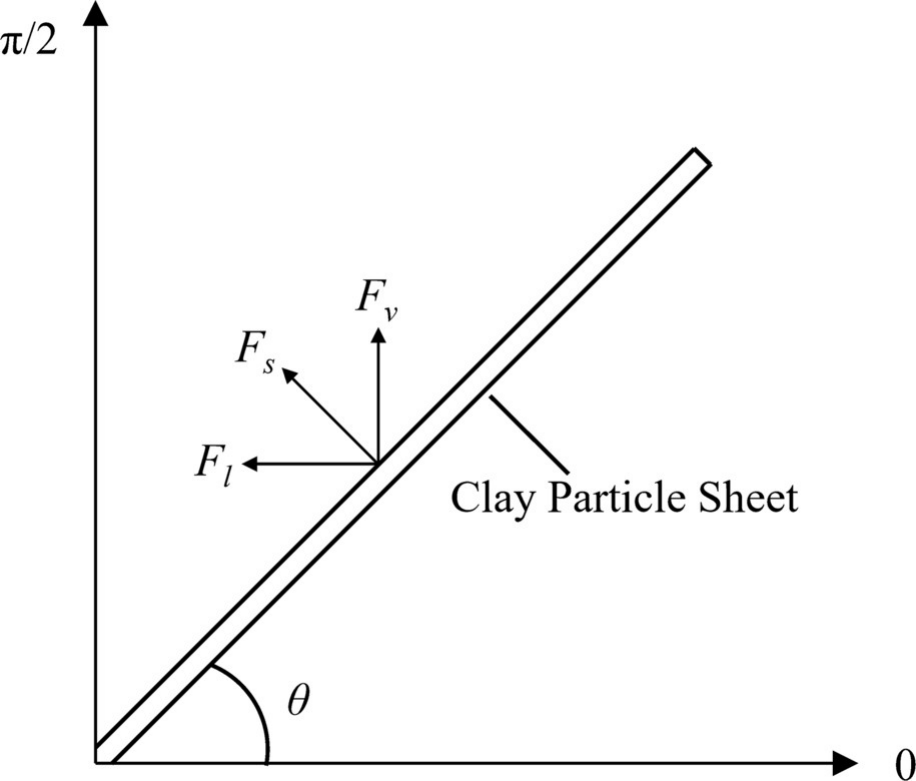
Diagram of the particle angle and the transverse and vertical expansion force.

The SEM images were processed using MATLAB software in Guangdong Major Infrastructure Safety Engineering and Technology Research Centre at Sun Yat-sen University (Fig 2). Image processing was performed in two steps. The first step was preprocessing and binary processing, and the second was particle boundary treatment. The purpose of step 2 was to study the orientation of particles under the influence of ions and water. The samples were placed on a circular table in a fixed direction to facilitate the comparability of the SEM results, and the images were further processed using an image-processing toolbox [26] by setting the range of particle orientation angles (*θ*) from 0~*π*/2. Combined with the model derived in Fig 2 and Section 2.1, the micromodel of the electric double layer of red-weathered expansive soil was analyzed. Three representative images of the thirty images of red-bed weathered expansive soils were processed through preprocessing, particle calibration, particle orientation data extraction, and first-order exponential function fitting (Fig 3). The data processing method used for particle orientation data extraction adopted the drawing method, and the particle orientation was calculated using a combination of the methods used to produce Figs 2 and 4. *R*^2^ is approximately 0.85 in each case. Data processing was performed using a combination of the drawing method and the least squares method.

The probability distribution function of grain orientation for expansive soils can be obtained based on Eq 16.

$$f(\theta )=ae^{\left(-\theta /b\right)}+c,$$ (16)

where *a*, *b*, *c* are constants greater than 0. According to the definition of probability density, the following equation can be obtained:

$$\int_{0}^{\pi /2}f(\theta )dx=\int_{0}^{\pi /2}\left(ae^{-(\theta /b)}+\text{c}\right)dx=1.$$ (17)

The relationship among the constants *a*, *b*, and *c* is found by solving Eq 17:

$$a=\frac{1-c\pi /2}{b(1-e^{-(\pi /2b)})}.$$ (18)

Substituting Eq 18 into Eq 16, the following equation can be obtained (0 < *θ* ≤ π/2):

$$f(\theta )=\frac{1-c\pi /2}{b\left(1-e^{-(\pi /2b)}\right)}e^{-(\theta /b)}+c.$$ (19)

Assuming that the angle between the particle or layer surface and the horizontal direction is *θ* (Fig 4) and the range of values is 0~π/2, the vertical component (*F*_*v*_) and the transverse component (*F*_*l*_) of the expansion force (*F*_*s*_) can be expressed as

$$F_{v}=F_{s}\cos\;\theta \;\text{and}$$ (20)

$$F_{l}=F_{s}\sin\;\theta .$$ (21)

According to the micromechanics of the composites, the average volume of physical quantities in the representative units of composites *Ᾱ* is equal to their average volume in the composites. That is,

$$\bar{A}=\frac{1}{v}\int Adv.$$ (22)

Assuming the soil is composed of *m* phase clay particles and that the volume of the *i* phase is *v*_*i*_, the vertical component of the expansive force for the whole soil body can be defined as

$$\begin{array}{l} F_{v}=\frac{1}{v}\int_{v}F(x)dv=\frac{1}{v}\sum_{r=1}^{m}\int_{v_{i}}F(x)dv=\sum_{i=1}^{m}\frac{v_{i}}{v}\frac{1}{v_{i}}\int_{v_{i}}F(x)dv\\ =\sum_{i=1}^{m}\frac{v_{i}}{v}F_{i}=\sum_{i=1}^{m}c_{i}F_{i} \end{array},$$ (23)

where $c_{i}=\frac{v_{i}}{v}$, $\sum_{i=1}^{n}c_{i}=1$, and $F_{i}=\frac{1}{v_{i}}\int_{v_{i}}F(x)$.

By replacing *c*_*i*_ in Eq 23 with the orientation probability function of soil particles *f*(*x*) and substituting it into *F*_*i*_ = *F*_*si*_ cos *θ*, we can obtain:

$$F_{v}=\sum_{i=1}^{m}f(x)F_{si}\cos\;\theta .$$ (24)

Assuming that the orientation distribution of particles is continuous, the integral of Eq 24 is written as

$$F_{v}=\int_{0}^{\pi /2}F_{s}\;\cos\;\theta \;f(\theta )d\theta =\int_{0}^{\pi /2}F_{s}\;\cos\;\theta \left(\frac{1-c\pi /2}{b(1-e^{-(\pi /2b)})}e^{-(\theta /b)}+c\right)d\theta .$$ (25)

Similarly, the integral form of the lateral expansive force of the soil is

$$F_{l}=\int_{0}^{\pi /2}F_{s}\;\sin\;\theta \;f(\theta )d\theta =\int_{0}^{\pi /2}F_{s}\;\sin\;\theta \left(\frac{1-c\pi /2}{b(1-e^{-(\pi /2b)})}e^{-(\theta /b)}+c\right)d\theta .$$ (26)

By calculating Eqs 25 and 26, the expressions for the vertical and transverse expansive force components of soils can be obtained:

$$F_{v}=\left(\frac{\left(1-c\pi /2)(1+be^{-(\pi /2b)}\right)}{\left(b^{2}+1)(1-e^{-(\pi /2b)}\right)}+c\right)F_{s};$$ (27)

$$F_{l}=\left(\frac{\left(1-c\pi /2)(b-e^{-(\pi /2b)}\right)}{\left(b^{2}+1)(1-e^{-(\pi /2b)}\right)}+c\right)F_{s}.$$ (28)

Therefore, by combining Eqs 15, 27 and 28, an electric double-layer micromodel of expansive soil with hydration force and soil-particle direction distribution can be obtained.

### 2.3. Analysis of expansion-control factors of expansive soil based on a double electric layer micromodel

The micromodel of the electric double layer of expansive soil developed in this study takes into account the influence of expansive anisotropy and intergranular expansive force. According to Eq 25, the higher the percentage of *θ*-oriented soil particles is among the total soil particles, the greater is the corresponding probability density value *f*(*θ*), the greater is the vertical expansive force, and the higher is the expansion potential. Although the magnitude of the soil-particle orientation *θ* is related to the direction of the vertical expansive force (that is, the direction of the maximum expansive deformation of the soil), *θ* has little effect on the total expansive potential of the soil. According to previous experimental studies on the microstructure and expansive potential of expansive soils [27], the surface–surface aggregates in expansive soils generally overlap and are arranged in parallel. This arrangement determines the direction of expansion, while the volume change in expansive soils dominated by surface–surface aggregates is much larger than that in expansive soils dominated by edge–surface aggregates after immersion. Therefore, the soil-particle orientation distribution is one of the critical parameters in the microexpansion model of expansive soil.

Eq 15 shows that the intergranular expansive force consists of an electric double-layer repulsive force, a hydration force, and a van der Waals force. According to Eqs 12 and 13, the surface potential of the Stern layer, the surface charge density of clay particles, the diameter of hydrated cations, and the specific surface area (which affects the surface charge density) of expansive soil play a decisive role in the magnitude of the repulsive force and the hydration force of the electric double layer. Both the repulsive force and the hydration force increase with an increase in the specific surface area of the expansive soil per unit volume. While the repulsive force of the double layer increases with an increase in the absolute value of the surface potential of the Stern layer, the diameter of the hydrated cations has a significant effect on the hydration force. In addition, the hydration force increases as the charge density of the Stern layer and the radii of the hydrated ions increase. Eq 14 shows that although the van der Waals force is mainly related to the thickness of the soil particles, it has little influence on the overall expansion force. Therefore, the intergranular expansive force of expansive soils is largely determined by the surface potential of the Stern layer, the surface charge density of clay particles, the diameters of the hydrated cations, and the specific surface area of the expansive soil.

The influence of specific surface area on the expansive properties of expansive soils has already been studied extensively [28]. The specific surface area of expansive soil depends mainly on the soil’s content of 2:1 expansive clay minerals. Type 2:1 clay minerals consist of two silicon-oxygen tetrahedral wafers sandwiched within an aluminum-oxygen octahedral wafer to form a crystalline layer. A large specific surface area leads to a greater swelling potential. It is difficult to directly determined the effects of the surface potential of the Stern layer, surface charge density of clay particles, and diameter of hydrated cations on the expansive properties of expansive soils. However, these three factors are closely related to the ion concentration, ion type, and pH of the aqueous solution.

The influence of the directional distribution of particles on the swelling characteristics of expansive soil has been described in this section, but relevant experimental verification was not conducted. The following study used the relationship between ion concentration, ion type, pH, and the expansion rate of the aqueous solution to verify the effects of the surface potential of the Stern layer, the surface charge density of clay particles, and the diameter of hydrated cations on the expansive properties of expansive soils.

In general, the electric double-layer microscopic model established in this paper has some similarities to previous theoretical research results such as the simplified model of clay expansion over time in an unsaturated environment studied by El Yaakoubi et al. [13] and the free expansion elastoplastic model of bentonite studied by Navarro et al. [12]. However, previous research did not consider the effect of the resulting water force or the particle orientation. In addition, the electric double-layer microscopic model is consistent with the results of previous microscopic experiments and numerical calculations; thus, it bridges the gap in theoretical research and establishes a relationship between theory and practice. For example, Kamal et al. (2019) found through experiments that water and hydrochloric acid cause expansion of clay minerals such as kaolinite, illite and chlorite, causing damage to the formation [17]. Rahromostaqim et al. (2019) found through numerical calculations that ion type has a great influence on the expansion of clay minerals [19]. This paper fills the gap in theoretical research and establishes a relationship between theory and practice. Of course, the good results of comparisons with the results obtained in previous studies also demonstrate the reliability of the theoretical model established in this paper.

## 3. Macroscopic experimental verification of the microscopic model of the electric double layer of expansive soil

To validate the developed microscopic model of the electric double layer of expansive soil, a series of experimental studies were conducted.

### 3.1. Test procedure

The sample used in our macro experiment was also obtained from a red-bed slope beside the highway in Maoming City, Guangdong Province. The dry density of the soil was 1.53 g/cm^3^. Macrospecimens were prepared by pressing undisturbed soil particles or soil particles soaked in salt solution into specimens with a diameter of 61.8 mm and a height of 20 mm. The difference in weight between any two samples in a testing group was less than 0.2 g. We then incubated the samples in a drying oven for 8 hours to measure the moisture content and prepare for the test.

As the unloading expansion rate of expansive soil is an ideal method for measuring the expansive potential of expansive soil, the unloading expansion rate test of expansive soil was conducted in this study using the WZ-2 soil dilatometer at the Guangdong Major Infrastructure Safety Engineering and Technology Research Centre at Sun Yat-sen University. The range of the dilatometer is 10 mm, and its measurement accuracy is 0.01 mm. Because the ion concentration, ion type, and pH of rainwater vary, the following experimental schemes were adopted. This met the requirement for model validation outlined in Section 2.3; because the experimental conditions were limited and most particles were distributed in parallel, the influence of the orientation distribution of soil particles was not considered.

1. The effects of NaCl solutions at various concentrations (0.005, 0.05, 0.5, and 1 mol/L) on the expansibility of red-bed weathered expansive soils were tested to investigate changes in the expansibility of expansive soils with the surface potential of the Stern layer and the surface charge density of clay particles;
2. The effects of 0.01 mol/L NaCl solutions at various pHs on the expansibility of red-bed weathered expansive soils were tested to further investigate the effects of the surface potential of the Stern layer and the surface charge density of clay particles on the expansibility of expansive soil. The pH of the NaCl solution was adjusted to 3, 5, 7, and 9 by dilution with hydrochloric acid solution or by the addition of sodium hydroxide powder.
3. The effects of NaCl, KCl, CaCl_2,_ and MgCl_2_ solutions at 0.1 mol/L on the expansibility of red-bed weathered expansive soils were tested to investigate the effects of hydrated cations with different radii on the expansibility of expansive soils.

**Fig 5:**
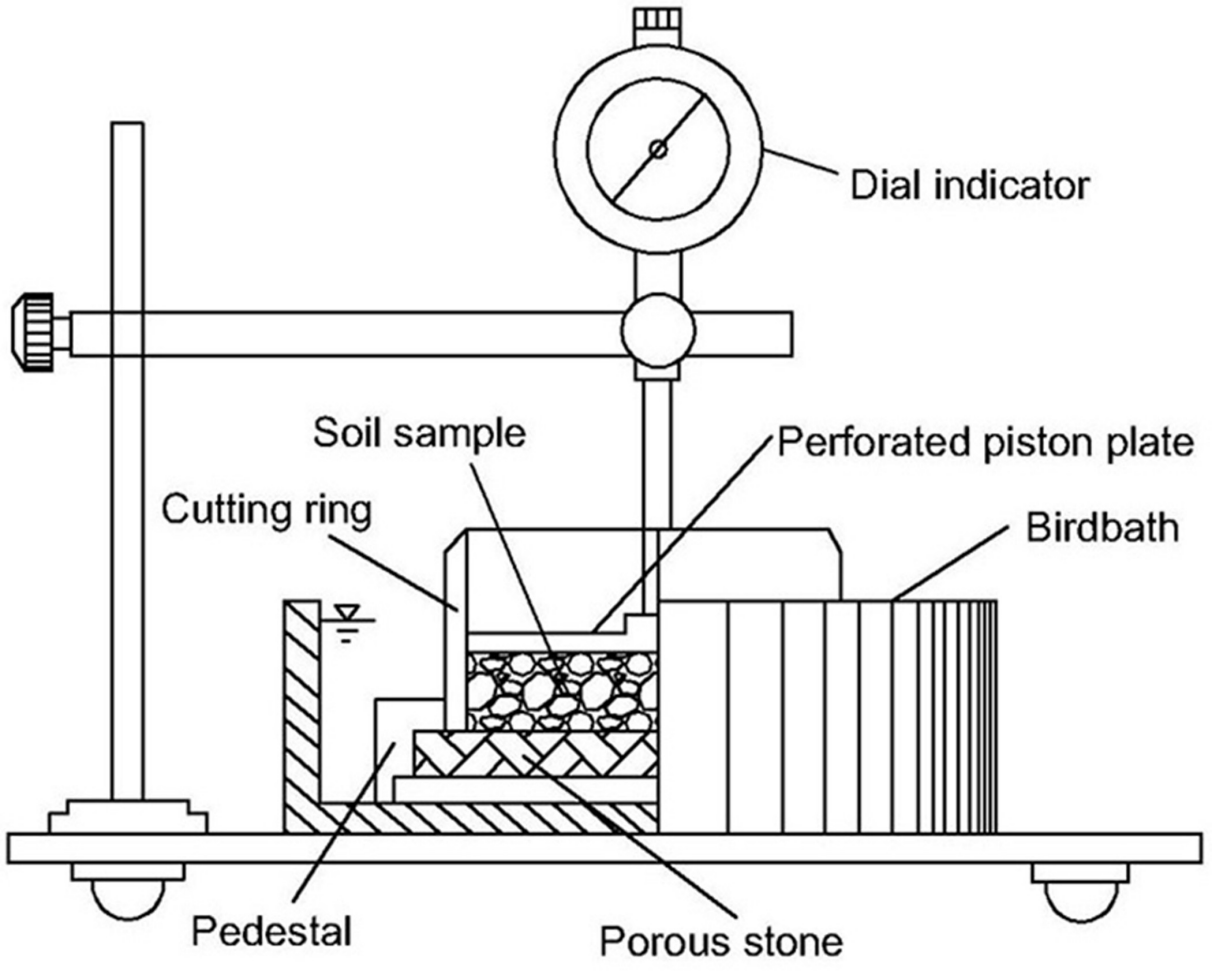
Experimental process profile.

A total of 12 groups of experiments are required for the above three experimental schemes. Three parallel experiments were conducted for each group. First, the solutions required to conduct the experimental scheme were prepared. Then, the blunt end of the cutting ring cutting through the soil sample was placed on the porous stone fixed on the base of the soil dilatometer, and the perforated piston plate was placed on the upper surface of the sample (Fig 5). The dial of the dial indicator was recalibrated and aligned with the perforated piston plate. Finally, the birdbath was filled with the specified solution, maintaining the water level 5 mm above the soil sample. Dial indicator readings were taken at 5, 10, 20, 30, 60, and 120 minutes after the experiment began. The dial indicator readings were then recorded every 2 hours until the difference between readings within 6 hours of each other did not exceed 0.01 mm; at that point, the experiment was terminated. The experimental data were analyzed by the tabulation method and the drawing method.

### 3.2. Experimental results and analysis

#### 3.2.1. Effect of NaCl solutions at different concentrations on the expansibility of soil samples

**Fig 6:**
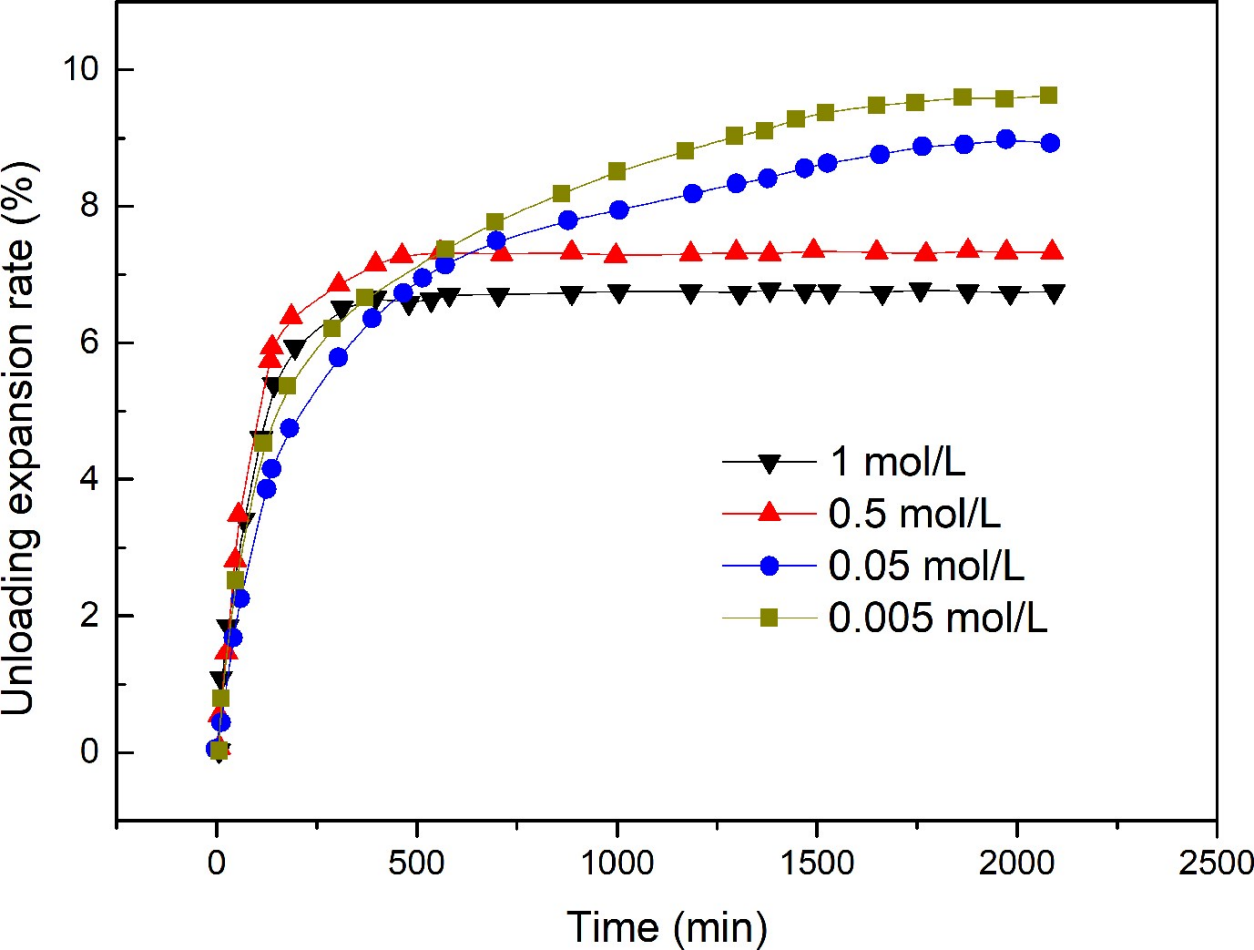
Time-dependent unloading expansion rate of expansive soils containing different concentrations of NaCl.

**Fig 7:**
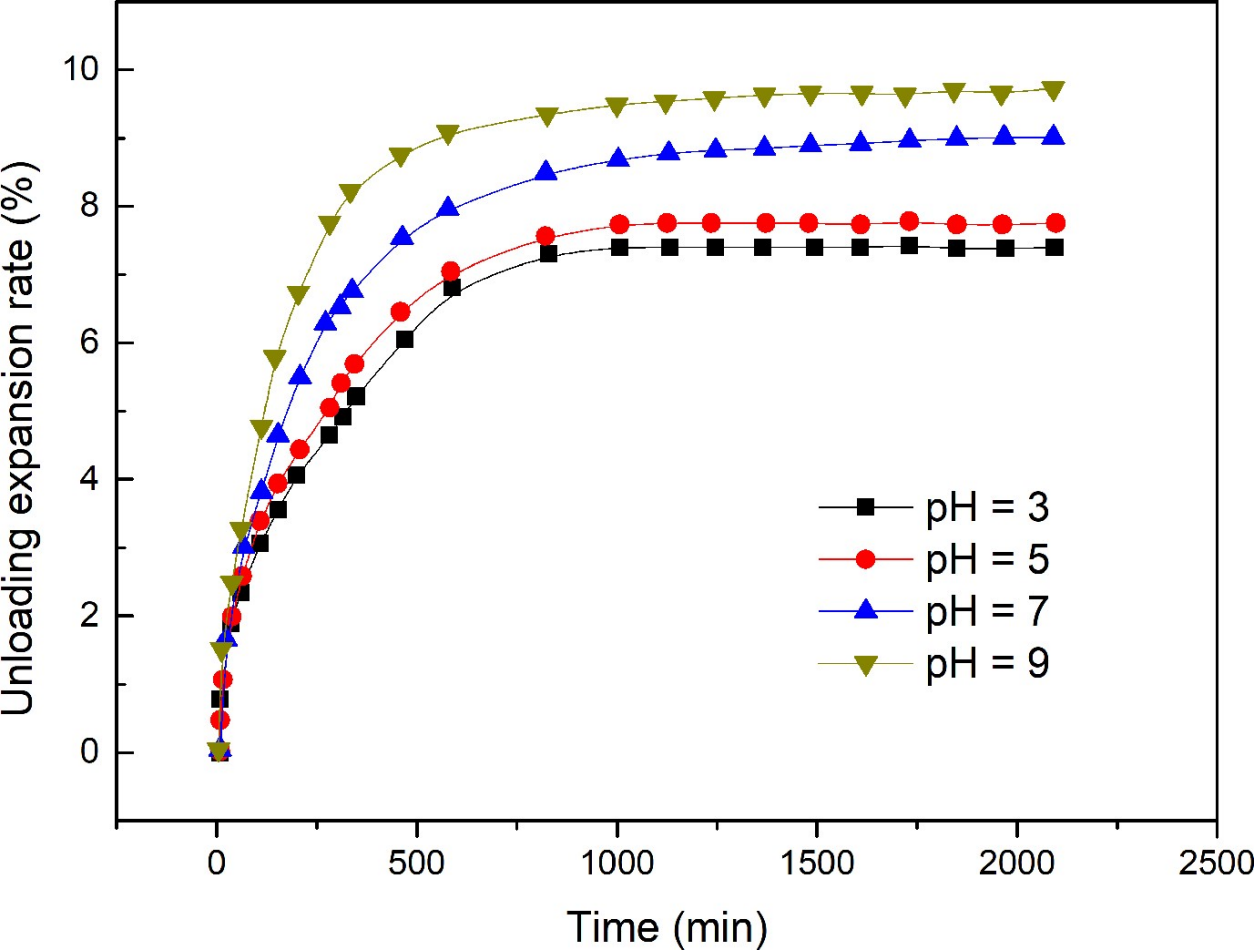
Time-dependent unloading expansion rate of expansive soils at different soil pH values.

**Fig 8:**
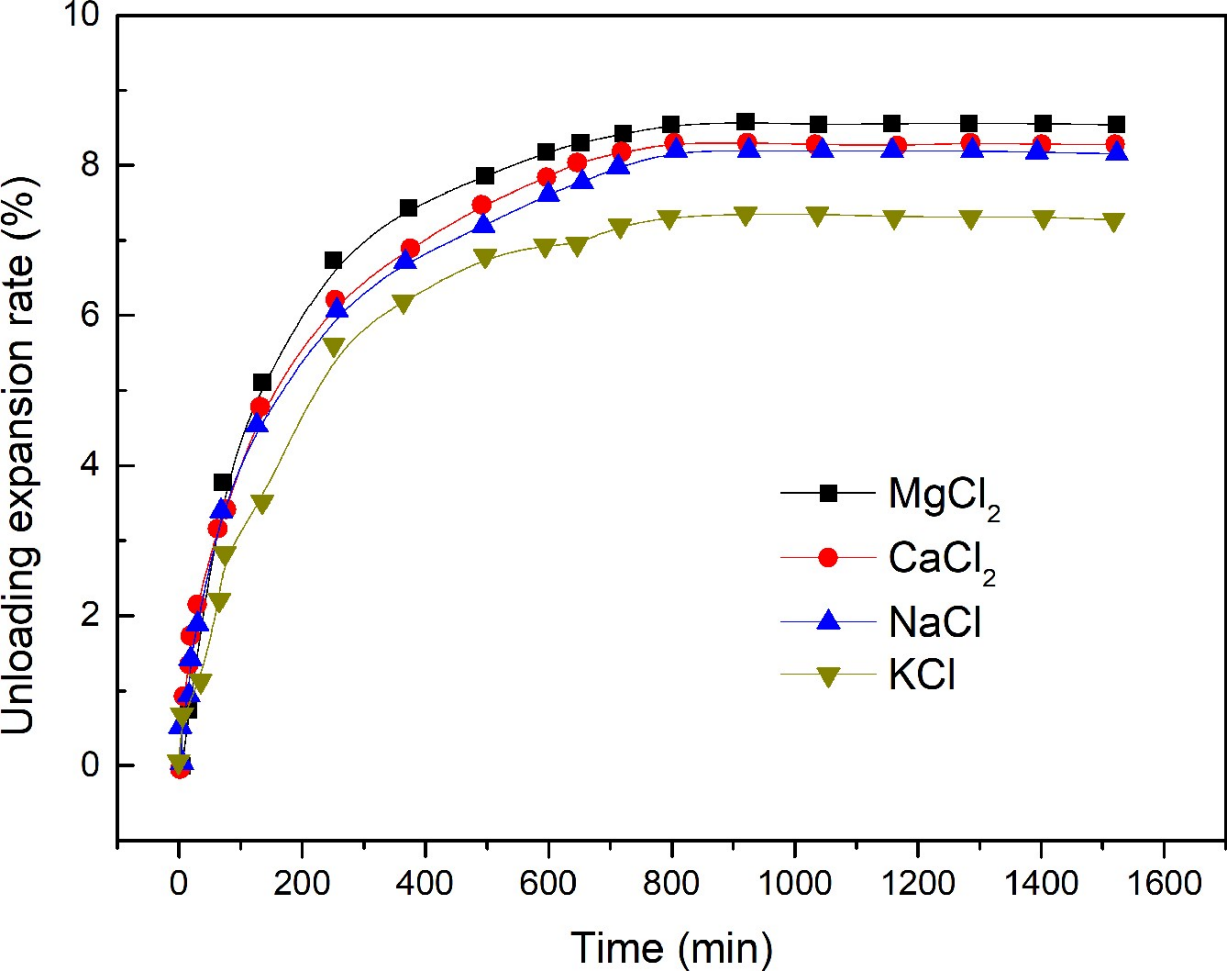
Time-dependent unloading expansion rate of expansive soils containing different types of ions.

The test results for the unloading expansion rate of soils containing NaCl at four different concentrations are shown in Table 2, and the time-dependent unloading expansion rate is shown in Fig 6. The data shown in Table 2 and Fig 6 are the mean values of the three parallel experiments, and the experimental error is within 5% (the data presented in Table 3, Fig 7, Table 4 and Fig 8 are also treated similarly).

**Table 2:** Unloading expansion rates of expansive soil at different NaCl concentrations.

NaCl concentration (mol/L)	0.005	0.05	0.5	1
**Unloading expansion rate (%)**	9.66	8.98	7.36	6.78

**Table 3:** Unloading expansion rates of expansive soil at various pH values.

pH value	3	5	7	9
**Unloading expansion rate (%)**	7.40	7.77	9.04	9.71

**Table 4:** Unloading expansion rates of expansive soils containing different types of ions.

Ion type	K^+^	Na^+^	Ca^2+^	Mg^2+^
**Unloading expansion rate (%)**	7.34	8.25	8.33	8.55

The experimental results show that the unloading expansion rate of red-bed weathered expansive soils decreases with increasing NaCl concentration. A higher concentration of NaCl reduces the time needed for the expansive soil to reach stable expansion. This is because the concentration of ions in the solution affects the concentration of hydrated cations in the diffusion layer on the surface of soil particles. As a result, the surface electric potential and the charge density of the soil particles will change and finally affect the expansion characteristics of the expansive soil. Specifically, a high concentration of cations forces more hydrated cations into the Stern layer, resulting in an increase in the charge density of the Stern layer and decreases in the charge density of the diffusion layer and in the surface potential of the Stern layer. In addition, Fig 6 shows that there is a significant increase in the unloading expansion rate at the initial stage of deformation because the hydration force increases with increasing charge density in the Stern layer. However, due to the short range of the hydration force, the hydration force disappears when a certain distance is reached; thereafter, the double electric layer plays a dominant role. When the solution-particle system reaches equilibrium, the presence of NaCl at a higher concentration results in lower final expansion of the soil. The expansion deformation is the result of the combined action of the hydration force and the double-layer repulsive force.

#### 3.2.2. Effect of pH on the expansibility of soil samples

The test results showing the unloading expansion rate of expansive soils containing NaCl at four different pH values are shown in Table 3, and the time-dependent unloading expansion rates of expansive soils at different pH values are shown in Fig 7.

Fig 7 shows that the time-dependent unloading expansion rate of expansive soils decreases as the soil pH decreases because the surfaces of the clay particles in expansive soil are negatively charged [29, 30]. The increase in the charge density of the clay particle surface and in the absolute value of the surface potential of the Stern layer leads to an increase in expansive deformation of the soil. According to the electric double-layer model of expansive soil, an increase in negative electricity on the surface of the clay minerals increases the hydration force and the electric double-layer repulsive force–that is, the expansive force. The experimental results are consistent with the model predictions.

#### 3.2.3. Effect of different ion solutions on the expansibility of soil samples

The test results for the unloading expansion rate of expansive soils containing four different types of ions are shown in Table 4, and the time-dependent unloading expansion rate of expansive soils in different types of ionic solutions is shown in Fig 8.

Table 4 indicates that for soils that have the same ion concentrations, the expansion rate of expansive soil, in descending order, is Mg^2+^ > Ca^2+^ > Na^+^ > K^+^. As the radius and capacity of cationic hydration, in descending order, are Mg^2+^ > Ca^2+^ > Na^+^ > K^+^, the unloading expansion rate of the expansive soil increases with an increase in the ion radius and capacity of cationic hydration. This is also consistent with the results obtained using Eq 12 in the microscopic model.

In general, the analysis presented in the above three sections is consistent with that in Section 2.3. This proves the reliability of the experimental results from the microscopic point of view. The results regarding the effects of ion concentration, pH and ion type on the expansibility of soil, clay and clay minerals are consistent with those of previous studies. For example, Tang et al. (2020) found that with increasing ion concentration, the contribution of expansion to soil deformation increased significantly [31]. Jiang et al. (2016) found that increasing the soil pH affected the stability of clay expansion and deformation [32]. Du et al. (2020) found that different cation types have different effects on the expansion characteristics of clay minerals [33]. The reliability of the experimental results is again proven at the macroscopic scale. To some extent, the model can overcome the deficiencies of the traditional electrical double-layer model in considering hydration force and particle orientation. Moreover, through theoretical analysis and experiments, the microscopic expansive behavior of expansive soil particles is connected with the macroscopic expansive phenomenon of soil.

## 4. Conclusions

In this study, aiming to model the soil expansion characteristics of rainfall-induced red-bed shallow landslides based on the Gouy-Stern electric double-layer theory, a microscopic model of the electrical double layer of red-bed weathered soil that considers the hydration force and particle orientation was established. The model combines the characteristics of soil and the influence of water on soil in the expansion model, thereby solving the problem that previous research on the expansion mechanism of expansive soil did not consider the influence of hydration and particle orientation.

The effects of three variables, namely, cation concentration, pH and cation type, on the expansion rate were studied using the unloading expansion rate test of weathered soil of red-bed slopes in South China. The results prove the reliability and feasibility of the microscopic model of the electrical double layer of red-bed weathered soil considering hydration force and particle orientation.

This study also provides a theoretical basis for analysis of the mechanism and prevention of rainfall-induced red-bed landslides, especially in South China, where rainfall is relatively high and red beds are widely distributed.
